# Back to the future: a very brief history of brief interventions

**DOI:** 10.1186/1940-0640-7-S1-A24

**Published:** 2012-10-09

**Authors:** Jim McCambridge, John Cunningham, Kypros Kypri

**Affiliations:** 1 The London School of Hygiene & Tropical Medicine, University of London, London, UK; 2 Center for Addiction and Mental Health, Toronto, Ontario, Canada; 3 School of Medicine or Public Health, The University of Newcastle, Newcastle, Australia

## 

This presentation is part of a larger study regarding the history of ideas about drinking alcohol and how it may be influenced, focusing on the evolution of thinking about brief intervention (BI). The early history of the BI field is reviewed, and consideration is given as to how key ideas were developed and the contemporaneous influences upon those ideas. Ideas about what constitutes BI and the nature and prominence of the facilitation of self-change have changed over time. Three seminal studies are reviewed in depth, comprising the first trial, the first review, and the most cited BI study. It is possible to anticipate future trends in BI research by examining the past. The development of the internet has important implications for BI, which, to some extent, calls upon earlier ways of thinking.

